# A Multi-Rheology Design Method of Sheeting Polymer Extrusion Dies Based on Flow Network and the Winter–Fritz Design Equation

**DOI:** 10.3390/polym13121924

**Published:** 2021-06-10

**Authors:** Amin Razeghiyadaki, Dongming Wei, Asma Perveen, Dichuan Zhang

**Affiliations:** 1 Department of Mathematics, School of Sciences & Humanities, Nazarbayev University, Kabanbay Batyr 53, Nur-Sultan 0100000, Kazakhstan; amin.razeghiyadaki@nu.edu.kz; 2 Department of Mechanical & Aerospace Engineering, School of Engineering & Digital Sciences, Nazarbayev University, Kabanbay Batyr 53, Nur-Sultan 0100000, Kazakhstan; asma.perveen@nu.edu.kz; 3 Department of Civil & Environmental Engineering, School of Engineering & Digital Sciences, Nazarbayev University, Kabanbay Batyr 53, Nur-Sultan 0100000, Kazakhstan; dichuan.zhang@nu.edu.kz

**Keywords:** polymer processing, sheet die design, manufacturing process design, coat-hanger die, modeling, rheology, constant shear-rate die, non-Newtonian fluids

## Abstract

In the polymer sheet processing industry, the primary objective when designing a coat-hanger die is to achieve a uniform velocity distribution at the exit of the extrusion die outlet. This velocity distribution depends on the internal flow channels of the die, rheological parameters and extrusion process conditions. As a result, coat-hanger dies are often designed for each polymer based on its individual rheological data and other conditions. A multi-rheology method based on a flow network model and the Winter–Fritz equation is proposed and implemented for the calculation, design and optimization of flat sheeting polymer extrusion dies. This method provides a fast and accurate algorithm to obtain die design geometries with constant wall-shear rates and optimal outlet velocity distributions. The geometric design when complemented and validated with fluid flow simulations could be applied for multi-rheological fluid models such as the power-law, Carreau–Yasuda and Cross. This method is applied to sheet dies with both circular- and rectangular-shaped manifolds for several rheological fluids. The designed geometrical parameters are obtained, and the associated fluid simulations are performed to demonstrate its favorable applicability without being limited to only the power-law rheology. The two such designed dies exhibit 32.9 and 21.5 percent improvement in flow uniformity compared to the previous methods for dies with circular and rectangular manifolds, respectively.

## 1. Introduction

One of the most important processes in the polymer processing industry is the extrusion of sheets. Coat-hanger dies are widely used in the polymer processing industry for the production of sheets and films. The desired design provides a uniform polymer sheet with constant thickness at the exit of the die. Uniform thickness only can be achieved when the polymer melt exits the die uniformly. Therefore, the design of a die can be accomplished by evaluating the design parameters that affect the velocity distribution at the exit of the die and by optimizing these parameters to obtain the desired uniform outlet velocity distribution. The latter depends on the rheological properties of the polymer melt entering the die, the process conditions such as flow rate and the internal geometry of the flow distribution in the manifold, as well as in the slit [1]. Therefore, finding the optimized internal design for sheeting extrusion die is of great importance.

In the literature, different designs of coat-hanger dies have been suggested and proposed by using analytical, numerical and experimental techniques with various degrees of uniform outputs. Reid et al. [2] presented an analytical method for the design of tapered coat-hanger dies for power-law fluids. A study by Winter and Fritz [3] proposed an analytical design method for circular- and rectangular-shaped manifolds for power-law fluids. Awe et al. [4] modified the Winter–Fritz method and applied it to rectangular-shaped manifolds with shortened die pre-land depth for the extrusion of degradable materials. Igali et al. [5] derived design equations of Casson fluids based on the Winter–Fritz model.

Other approaches for the design of extrusion dies include the integration of a computational fluid dynamics (CFD) method, such as finite volume or the finite element method with an optimization algorithm for the evaluation of the desired design parameters. Han and Wang [6] integrated a CFD simulation with a genetic algorithm for the optimization of velocity distribution and residence time of a tapered coat-hanger die. Lie et al. [7] proposed a modified finite element method, namely finite piece method, for the simulation of a tapered coat-hanger die. Their method proved to save up to 80% of the CPU time. Smith [8,9] presented a design methodology based on the finite element and the adjoint-variable optimization method for power-law, Carreau–Yasuda, Cross, Elis and Bingham fluids. An optimal geometry was obtained from their proposed methodology, although their results showed an insignificant dependency of optimal geometry on the fluid model. Lebaal [10,11,12] studied the capability of different optimization algorithms such as response surface method, Kriging interpolation, and the sequential quadratic method for the optimization of geometry and processing parameters. Razeghiyadaki et al. [13] developed a design method based on the response surface method and B-spline method for the optimization of die geometries, specifically for die profiles. However, the majority of previous studies have been focused on power-law fluids.

Another possible approach is devising a semi-analytical method, such as the flow network method, for the fast and accurate design of an extrusion die. A flow network analysis, also known as a hydraulic-electric circuit analogy, uses the conventional concept of electric circuit theory for an analysis of fluid flow problems [14]. The literature demonstrates [14,15] the application of this method to design coat-hanger dies with general rheology. Michaeli et al. [16] combined the finite element method with the network theory to find the optimum velocity distribution. They demonstrated this network as being optimal for the design of rectangular- and circular-shaped manifold extrusion dies for Carreau–Yasuda and power-law fluids. Yilmaz and Kadikkopru’s model was successful in identifying only geometrical parameters in manifold (i.e., height of the manifold).

Analytical methods tend to oversimplify the extrusion die problem, and these are limited to simple rheological models such as the power-law [3] or Elis model [17]. However, the power-law model is not applicable in a situation with very low shear rates [18,19]. More sophisticated rheology models such as Carreau–Yasuda represents the apparent viscosity at low shear rates more accurately. Nevertheless, analytical pressure-drop/flow-rate relation is not available for these rheology models. Since previous studies such the one of Liu and Liu [17] require an analytical pressure-drop/flow-rate relation, these methods have limited applicability. Therefore, there is always a need for a single design method that satisfies multiple generalized rheology fluids. Secondly, CFD-based design methods require numerous simulations [16]; as a result, in term of computational time, these methods are computationally expensive. In addition, due to the high cost of die block fabrication, a trial-and-error approach for the design of extrusion dies is very costly. On the other hand, numerical and analytical methods are either computationally expensive or lack accuracy due to unrealistic assumptions. Hence, a fast and accurate model for finding the optimized internal geometry of extrusion dies while being flexible in term of rheology is of great importance. In this study, a computationally fast semi-analytical method is established by constructing a flow network model.

Based on the circuit method introduced in [14] for fluid networks and the work shown in [15] for the application of such methods to coat-hanger die design, a multi-rheology design method is proposed and implemented for sheeting die design for general rheology fluids. This method has been demonstrated to be computationally efficient and effective through the design cases of dies with rectangular and circular manifolds. The obtained results are validated and compared against the existing results in the literature. Yet, it is shown to have an extended capability for finding both geometrical parameters of the manifold and the slit region for a uniform outlet velocity distribution. In addition, it can be adapted to any rheological model with few modifications. The method utilizes a modified discretized form of the Winter–Fritz equation on a flow network, which is considered a novel extension and contribution to the circuit network method for die design. Moreover, this method enforces additional uniform velocity distribution constraints, resulting in a significant improvement in the velocity distribution without conducting full-fledged CFD analyses on the manifold.

## 2. Methodology

**Figure 1:**
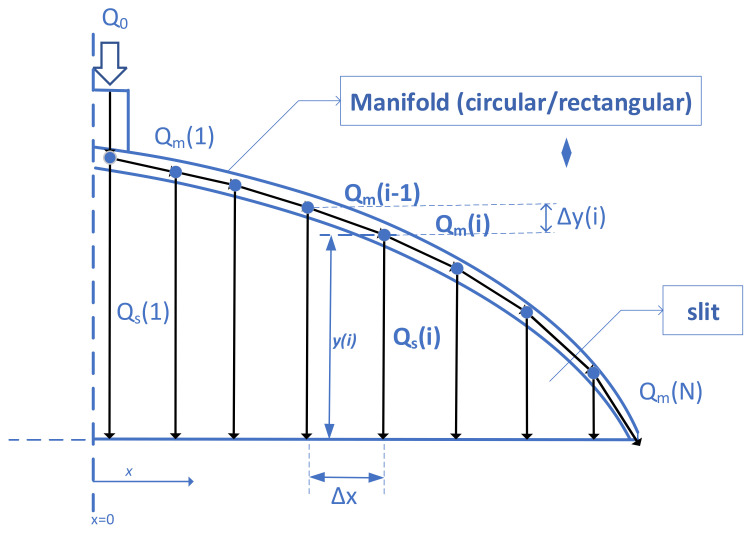
Schematic of a sheeting extrusion die and the corresponding flow network.

As shown in Figure 1, a coat-hanger die consists of two parts: the slit and the manifold. The design of a die can be reduced to the evaluation of the curve for the manifold and the variance in the cross-sectional area along the manifold length. The manifold cross-section area determines the flow resistance at various locations along the curve of the manifold. In addition, the manifold cross-section area specifies how much of the polymer gets to be distributed into the slit at each point along the curve. In the case of a manifold with a circular cross-section, radius (R) is considered the geometrical parameter, while in the case of a rectangular manifold, the height of the manifold (H) is considered the geometrical parameter of the manifold, and each determines the flow distribution. Moreover, the die profile, which is also known as the preland area, determines flow resistance in the slit. The goal of the die design is to find an optimized flow distribution manifold curve that yields constant flow rates at the die exit through the slit region. To demonstrate the proposed flow network method, circular and rectangular cross-sections of manifolds are selected as examples. With slight modifications, the cross-sectional shapes can be modified between these two or any other desired geometry, which are not considered here.

Due to the symmetry in geometry, only half of the die is modeled. The die is divided into a finite number (N) of segments along the *x*-axis. The proposed method can be utilized for a simple prediction of the flow distribution at the outlet or the design of the extrusion die by withholding certain constraints. In both cases, following assumptions are made for the proposed method:

1. Isothermal incompressible flow
2. Streamlined flow
3. Steady and fully-developed flows in both slit and manifold
4. Uniform pressure at the exit of the slit
5. Unidirectional flow in both manifold and slit network segments (ignoring traverse flow in the manifold)

In a previous study, it was observed that, due to the laminar nature of the flow in an extrusion die, the flow field is streamlined, and each fluid particle’s path does not cross any other particle’s path [13]. In addition, the flow field can be discretized as a summation of a finite number of separate flow lines, as shown in Figure 1. Polymer melt with flow rate of *Q*_0_ enters at the die. At each node of the manifold, a portion of polymer flow goes into the slit, while the rest is sent forth to the next manifold segment. At each segment, flow rates inside the slit and the manifold are denoted by *Q*_s_(*i*) and *Q_m_*(*i)* (*i* = 1 to *N*), respectively. The vertical distance between the manifold and the die exit is denoted by *y*(*i).* The vertical and horizontal distances between two adjacent manifold by *Q_s_*(*i*) segments are Δ*y* and Δ*x*, respectively.

### 2.1. Calculation of the Flow Distribution

When the geometry of the die is known, the model can predict the flow distribution at the die exit. The conservation of mass for each node on the manifold (see Figure 1) gives the following:

(1) $$Q_{m}\left(i-1\right)=Q_{m}\left(i\right)+Q_{s}\left(i\right)\;\;\;\;\;\;\;\;\;\;\;\;\;\;1\leq i\leq N$$

with the condition:

(2) $$Q_{m}\left(0\right)=Q_{0}$$

It is known that the Hagen–Poiseuille equation relates pressure drop and flow rate in a pipe or a duct [*Q* = *Q*(Δ*P*)]. For illustration, Hagen–Poiseuille equations of a power-law fluid are given as follows:

(3) $$Q_{s}\left(i\right)=\frac{2\left(2n+1\right)}{n\Delta xh_{s}^{2}}{\left(\frac{\Delta P_{s}\left(i\right)}{2k\;y\left(i\right)}\right)}^{1/n}$$

(4) $$Q_{m}\left(i\right)=\frac{n\pi R{\left(i\right)}^{3}}{3n+1}{\left(\frac{\Delta P_{m}\left(i\right)R\left(i\right)}{2k\Delta \zeta \left(i\right)}\right)}^{1/n}$$

(5) $$Q_{m}\left(i\right)=\frac{2\left(2n+1\right)}{nWH{\left(i\right)}^{2}}{\left(\frac{\Delta P_{m}\left(i\right)}{2k\Delta \zeta \left(i\right)}\right)}^{1/n}\;$$

where Equation (3) applies to the slit, Equation (4) applies to the circular-shaped manifold, and Equation (5) applies to the rectangular-shaped manifold.

**Figure 2:**
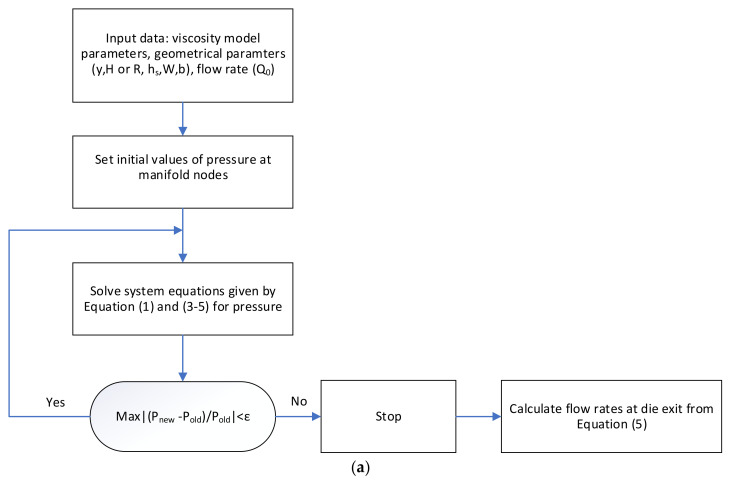

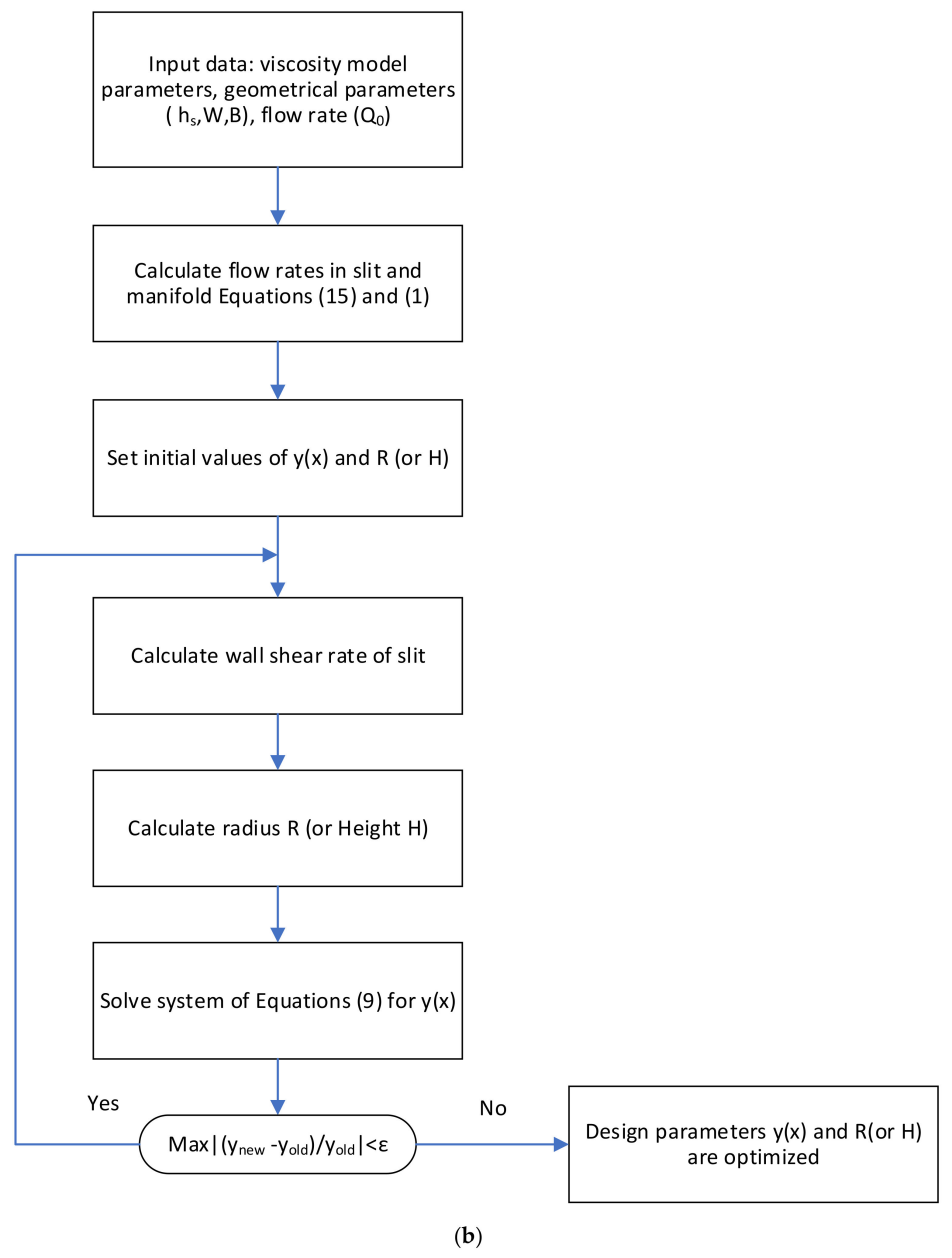
Flow charts for (**a**) flow distribution calculation and (**b**) proposed extrusion die design method.

By substituting Equation (3) and either Equation (4) or Equation (5) into Equation (1), a set of equations with the unknowns of pressure at the manifold segments is produced. Since the pressures at the exit of the slit are assumed to be equal to zero (atmospheric), pressures at the end of the slit are removed in Equation (3) and therefore, the only remaining unknowns are the pressure at manifold nodes. In summary, solving Equation (1) with the appropriate Hagen–Poiseuille equations, the pressure at the manifold nodes can be calculated. With calculated pressure at manifold nodes, the flow distribution at the exit can be calculated from Equation (3). The system of equations is numerically solved by the secant method. Figure 2a shows an algorithm for this method of calculation for flow distribution. Convergence criterion is set as:

(6) $$max\left|\frac{P_{new}\left(i\right)-P_{old}\left(i\right)}{P_{old}\left(i\right)}\right|<\varepsilon \;\;\;\;1\leq i\leq N$$

where convergence is achieved when *ε* is to 10^−4^.

### 2.2. Design of the Manifold Curve

A design procedure is required to find geometrical parameters for given process conditions and polymer melt rheology. Geometrical parameters defining an extrusion dies are radius *R*(*x*) (circular manifold) or height *H*(*x*) (rectangular manifold) and *y*(*x*), defined as the distance between the manifold and the die outlet at each *x* along the width of the die. For each segment shown in Figure 1, two equations are required. Besides the assumptions made for Equation (2), the following constraints are selected to determine the curve for the manifold:

1. Constant shear rates between the slit and the manifold and
2. Uniform velocity distribution at the exit of the slit.

The uniform shear-rate assumption gives the following:

(7) $$\gamma_{m}\left(R\;or\;H\right)=\gamma_{s}\left(y\right)$$

The uniform shear-rate dies satisfying the above constraints are also known as the Winter–Fritz dies [3], which satisfy the following differential equation:

(8) $$\frac{dy}{dx}=-{\left[{\left(\frac{{\left(\frac{dP}{dy}\right)}_{s}}{{\left(\frac{dP}{d\zeta }\right)}_{m}}\right)}^{2}-1\right]}^{-1/2}$$

for the determination of the curve of the manifold as it is a solution of *y*(*x*).

In Equation (8), *ζ* is the distance between two adjacent nodes along the manifold (*dζ^2^ = dx^2^ + dy^2^*). Subscripts m and s refer to the manifold and the slit, respectively. In a discretized finite difference form, Equation (8) can be written as follows:

(9) $$\left(y_{i+1}-y_{i}\right)=-\left(x_{i+1}-x_{i}\right){\left[{\left(\frac{{\left(\frac{dP}{dy}\right)}_{s}\left(i\right)}{{\left(\frac{dP}{d\zeta }\right)}_{m}\left(i\right)}\right)}^{2}-1\right]}^{-1/2}$$

The ratio of pressure gradients $\frac{{\left(\frac{dP}{dy}\right)}_{s}\left(i\right)}{{\left(\frac{dP}{d\zeta }\right)}_{m}\left(i\right)}$ in Equation (9) depends on both radius *R* (or height *H*) and *y*(*x*). Therefore, with known relations for wall shear rates and pressure gradients in the slit and the manifold, geometrical parameters *R* (or *H*) and *y(x)* can be calculated iteratively by solving Equations (7) and (9). For illustration, the wall shear rates and pressure gradients of a power-law fluid with a circular manifold are shown below (see [1] for similar formulas):

(10) $$\gamma_{w}\left(i\right)=\frac{2\left(2n+1\right)Q_{s}\left(i\right)}{n\Delta xh_{s}^{2}},1\leq i\leq N\;\mathrm{slit}\;$$

(11) $$\gamma_{w}\left(i\right)=\frac{\left(3n+1\right)Q_{m}\left(i\right)}{n\pi R{\left(i\right)}^{3}}1\leq i\leq N\;\;\;\;\mathrm{manifold}$$

(12) $${\left(\frac{dP}{d\zeta }\right)}_{m}\left(i\right)=\frac{\Delta P_{m}\left(i\right)}{\Delta \zeta \left(i\right)}=2k{\left(\frac{\left(3n+1\right)Q_{m}\left(i\right)}{n\pi R{\left(i\right)}^{\frac{3n+1}{n}}}\right)}^{n}$$

(13) $${\left(\frac{dP}{dy}\right)}_{s}\left(i\right)=\frac{\Delta P_{s}\left(i\right)}{\Delta y\left(i\right)}=2k{\left(\frac{2\left(2n+1\right)Q_{s}\left(i\right)}{n\Delta x\;h_{s}^{2}}\right)}^{n}$$

The substitution of Equations (10) and (11) in Equation (7) gives the following equation of the radius:

(14) $$R\left(i\right)={\left(\frac{3n+1}{2\left(2n+1\right)}\frac{Q_{m}\left(i\right)}{Q_{s}\left(i\right)}\Delta xh_{s}^{2}\right)}^{1/3}$$

The second constraint of the model states a uniform velocity distribution at the exit of the die. With this constraint, flow rates at the slit are as follows:

(15) $$Q_{s}\left(i\right)=\frac{Q_{0}}{N+1}\;\;\;\;1\leq i\leq N$$

With known flow rates of the slit, flow rates in the manifold can be obtained by the conservation of mass given in Equation (1).

Similarly, two systems of Equations (14) and (9) with the pressure gradient Equations (12) and (13) and flow rates from Equations (1) and (15) are solved by an iterative numerical scheme, such as the secant method. The convergence criterion is defined as follows:

(16) $$max\left|\frac{y_{new}\left(i\right)-y_{old}\left(i\right)}{y_{old}\left(i\right)}\right|<\varepsilon =10^{-4}\;\;\;\;1\leq i\leq N$$

Figure 2a shows the algorithm used for calculations.

### 2.3. Calculation of Wall Shear Rates and Pressure Gradients

The solution of the design Equation (8) requires information on shear rates at the walls of the manifold and the slit, and the pressure gradients respectively. This section provides a method for the evaluation of these values using a general non-Newtonian rheology equation:

(17) $$\mu =\mu \left(\gamma \right)$$

Most Poiseuille and shear-rate relations given in the literature have limited capacity to only one (generalized) non-Newtonian fluid, such as the power-law fluid [1,20,21].

Sochi, based on the Weissenberg-Rabinowitsch-Mooney-Schofield (WRMS) method, gives a relation for the wall shear rate of a fluid flowing between two parallel plates [18]:

(18) $$Q=\frac{2\Delta xh_{s}^{2}I_{pp}}{\tau_{w}^{2}}$$

where

(19) $$I_{pp}=\int_{0}^{\tau_{w}}\gamma \tau d\tau =\int_{0}^{\gamma_{w}}\left[\gamma^{3}\mu \frac{d\mu }{d\gamma }+\mu^{2}\gamma^{2}\right]d\gamma$$

(20) $$\tau_{w}=\mu \left(\gamma_{w}\right)\gamma_{w}=\frac{h_{s}\Delta P}{y\left(x\right)}$$

In these equations, *h*_s_, and ∆*x* are the thickness and width, respectively. From Equation (18), the wall shear rates can be calculated for given pressure gradient Δ*P*. Meanwhile, for a given flow rate there is no explicit relation for the calculation of the pressure drop Δ*P*. Therefore, an iterative numerical scheme is required. The same WRMS method has been adapted for the flow in a circular duct:

(21) $$Q=\frac{\pi R^{3}I_{c}}{\tau_{w}^{3}}$$

(22) $$I_{c}=\int_{0}^{\dot{\tau_{w}}}\gamma \tau^{2}d\tau =\int_{0}^{\dot{\gamma_{w}}}\left[\gamma^{4}\mu^{3}\frac{d\mu }{d\gamma }+\mu^{3}\gamma^{3}\right]d\gamma$$

(23) $$\tau_{w}=\mu \left(\gamma_{w}\right)\gamma_{w}=\frac{R\Delta P}{2\Delta \zeta }$$

where *∆ζ* and *R* are the length and radius of the manifold segment. Equations (18)–(20) give the wall shear rate in a slit segment, while Equations (21)–(23) give the wall shear rate in a circular manifold. The design curve *y*(*x*) is obtained by solving these equations and then substituting pressure gradients in Equation (9), respectively, for the polymer melt rheology models. It is important to notice that Equations (19)–(23) are valid for the general rheology model in the form of Equation (17). Therefore, by adapting them in the proposed method, the restriction and limitation of the traditional constant shear-rate dies applicable to the power-law fluids can be relaxed.

## 3. Results and Discussion

### 3.1. Validation

**Figure 3:**
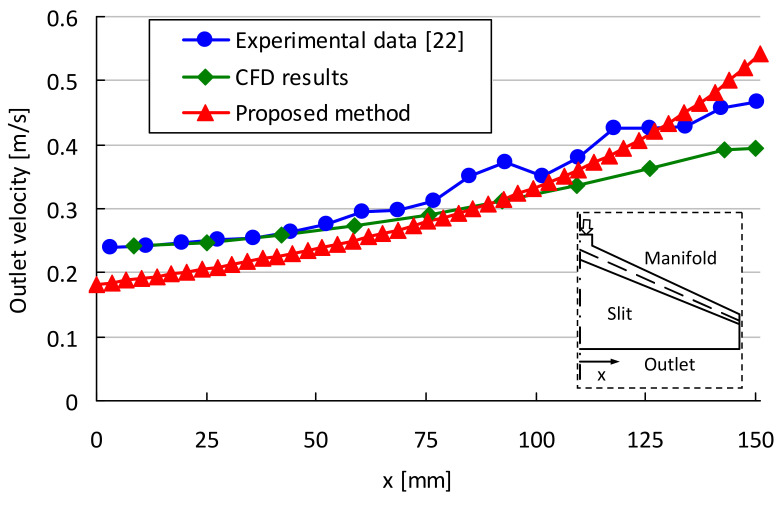
Validation of the model and its comparison with CFD and experimental data [22].

For validation of the proposed method, the fluid distribution at the exit of a die obtained from it is compared with the experimental data of Meng et al. [22] and with the results of a CFD analysis. Meng provides the flow distribution for a tapered extrusion die with power-law consistency factor (*k*) and index (*n*) of 0.799 Pa s^n^ and 0.696, respectively. Details of the process conditions and geometrical parameters can be found in Meng [22]. The flow distribution is calculated (Figure 2a) for the given geometry and process conditions of Meng’s tapered die. Figure 3 depicts the comparison of the velocity at the outlet for experimental data [19] and the CFD simulation performed on Ansys Fluent 19.1. As it can be seen, our method shows good agreement with the experimental data and the CFD simulation results. The main difference between the obtained results and the CFD could be due to the three-dimensional effects. In each segment, part of the flow enters the slit. Consequently, the flow field in the manifold is not completely unidirectional (assumption e), and the flow field in the manifold has a transverse component. In addition, the carboxymethyl cellulose (CMC) water solution used in Meng’s study has lower power-law consistency factor (*k*) than common polymer melts. As a result, the fluid flow of the CMC solution may enter the transitional flow regime, which in turn defies the stratified flow pattern in assumption b.

### 3.2. Sensitivity Analysis

**Figure 4:**
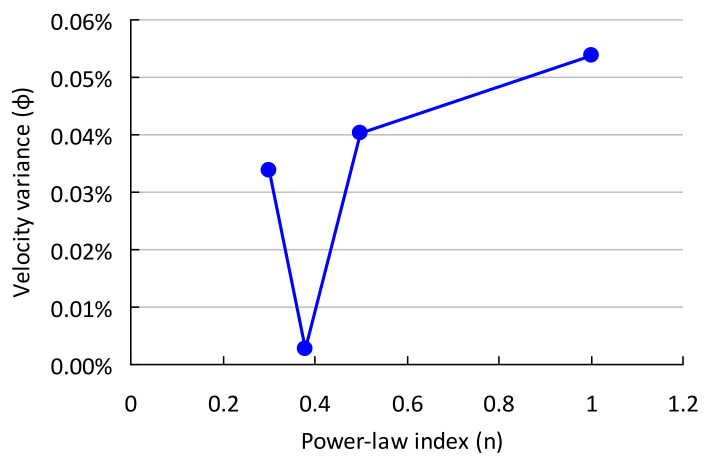
Sensitivity analysis of the model for a power-law fluid (*Q*_0_ of 5 × 10^−5^ m^3^/s, *h_s_* of 1.5 mm and *b* of 360 mm).

In the sensitivity analysis, the optimized design of a power-law fluid with input design parameters given in Table 1 and a power-law index of 0.38 is performed by the design methodology (algorithm in Figure 2b). Subsequently, for the obtained design profiles of *y*(*x*) and radius (*R*), the flow distribution is calculated (algorithm in Figure 2a) for fluids with different power-law index values. As discussed earlier, an extrusion die with a uniform velocity at the outlet is desired. Therefore, velocity variance is defined as follows:

(24) $$\varphi =\frac{\sum_{0}^{N}{\left(v_{i}-v_{ave}\right)}^{2}}{N}$$

where *φ, v_i_* and *v_ave_* are velocity variance, velocity at slit segment *i* and the average velocity of all segments. Figure 4 depicts the velocity variance for fluids with different values of the power-law index (n) running through a die whose geometries were optimized (*y*(*x*) and manifold radius *R*) for a power-law index (n) of 0.38. In Figure 4, the flow rate at entry *Q_0_*, slit height h_s_ and half of die width *b* are 5 × 10^−5^ m^3^/s, 1.5 mm and 360 mm, respectively. Since a uniform velocity is assumed in the design method (algorithm in Figure 2b), the velocity variance calculated from the proposed method (algorithm in Figure 2a) for the fluid with a power-law index (n) of 0.38 is nearly zero, since a uniform flow distribution is assumed (Equation (15)). However, this die geometry gives higher velocity variance for other fluids with different power-law indices. This observation shows that an optimized extrusion die design is dependent on the fluid rheological parameters.

### 3.3. Design of a Power-Law Fluid with Circular Manifold

Three rheological models are considered in this study as examples. Based on the constant shear-rate assumption, Winter and Fritz [3] provided a design model for a power-law fluid that flows in an extrusion die. Their model gives the following equations for the radius *R*(*x*) of the manifold and its profile curve *y:*

(25) $$R\left(x\right)=h_{s}{\left[\frac{\left(b-x\right)\left(1+3n\right)}{\pi h\left(1+2n\right)}\right]}^{\frac{1}{3}}$$

(26) $$y\left(x\right)=\frac{3Bb}{2}\left[\frac{\sqrt{1+g\left(x\right)}}{g\left(x\right)}-\frac{1}{2}\ln\frac{\sqrt{1+g\left(x\right)}-1}{\sqrt{1+g\left(x\right)}+1}\right]$$

where

(27) $$g\left(x\right)=({\left(R/h_{s}{)}^{2}-1\right)}^{-1}$$

(28) $$B=\frac{\pi h}{b}\frac{1+2n}{1+3n}$$

**Figure 5:**
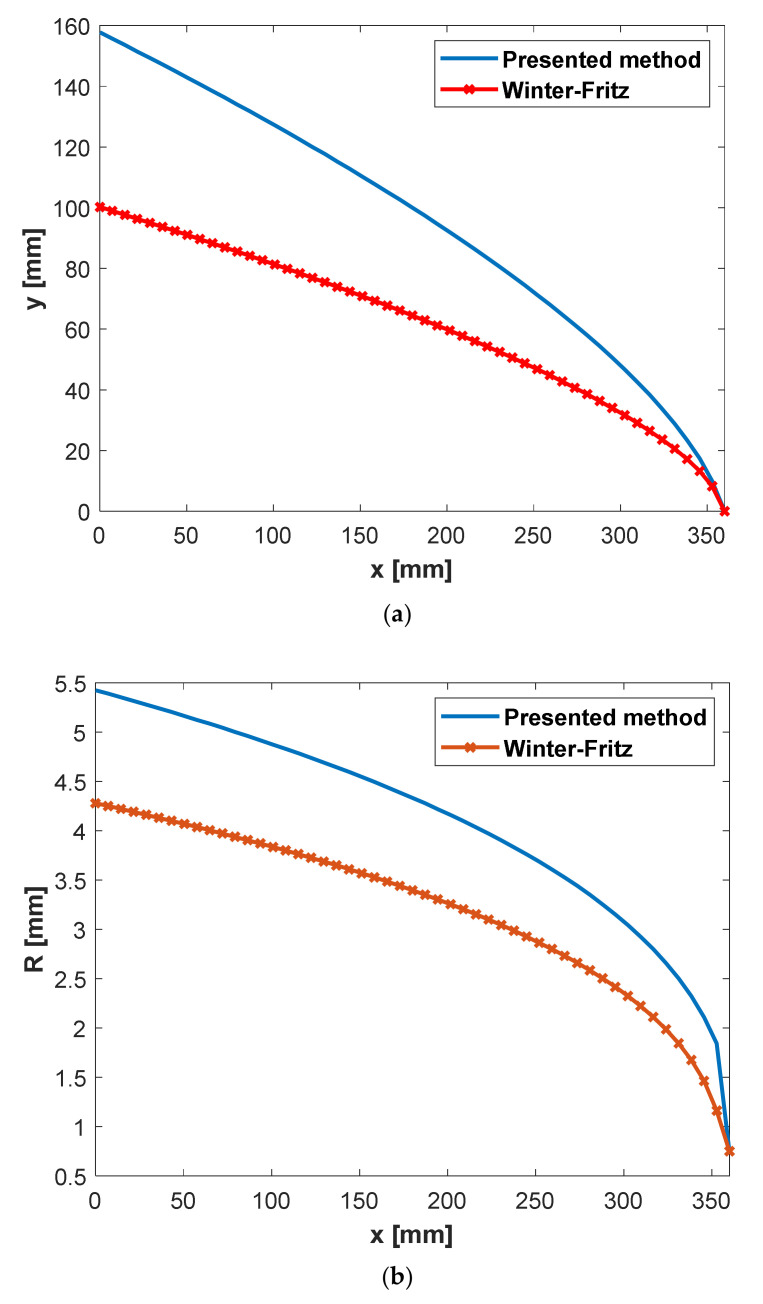
(**a**) Change of design parameter *y* with respect to *x* (**b**) Change of manifold radius *R* with respect to *x* for power-law polypropylene (parameters in Table 2) with a circular manifold.

Figure 5 shows two designs from Winter and Fritz and the proposed method for a power-law rheological model. The consistency factor and power-law index of the rheology model is given in Table 1. Input design parameters are also shown in Table 1. *y*(*x*) is a distance between the manifold and the die outlet, and R denotes the manifold radius. As is shown, the proposed method gives higher values for both *y*(*x*) and the manifold radius (*R*). Larger *y*(*x*) and manifold radius correspond to deeper die cavity and a larger cross-sectional area of the manifold, respectively. In addition to the shared assumption of a constant wall shear rate in both the Winter–Fritz and the proposed design methods, a new assumption of a uniform flow rate at the exit exerts an additional constraint on the proposed method. This new assumption provides a different profile for *y*(*x)* and the manifold radius (*R*) compared to the Winter–Fritz method.

The input of the design model is given in Table 2. After some preliminary calculations, the calculated results were found to be invariant for 50 or more number of segments (N).

In order to validate the performance of two available designs, a CFD package, Ansys Fluent 19.1, was used to show the flow distribution inside the die. Due to symmetry, only a quarter of the die is considered as the simulation domain. A python code was developed to build a mathematically accurate geometrical representation of die designs.

The CFD package of Ansys Fluent 19.1 solves Navier–Stokes equations via the finite volume method. Continuity and momentum equations for a fluid with density of *ρ* without body force are as follows:

(29) $$\nabla \cdot \left(\rho \vec{v}\right)=0$$

(30) $$\nabla \cdot \left(\rho \vec{v}\vec{v}\right)=-\nabla p+\nabla \cdot \bar{\tau }$$

where *v* is velocity, *p* is static pressure and *τ* is stress tensor.

The stress tensor is given by:

(31) $$\bar{\tau }=\mu \left[\left(\nabla \cdot \vec{v}+\nabla \cdot {\vec{v}}^{T}\right)-\frac{2}{3}\nabla \cdot \vec{v}I\right]$$

where *I* is the unit identity tensor and *µ* is viscosity.

**Figure 6:**
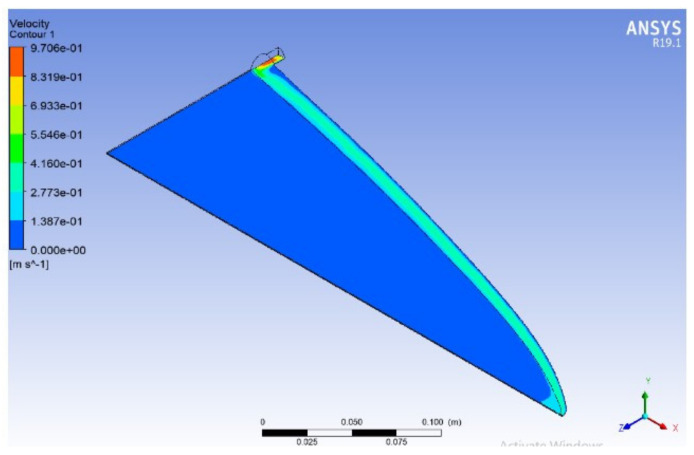
Velocity distribution of Winter–Fritz coat-hanger die design with a circular manifold (flow rate *Q_0_* = 5 × 10^−5^ m^3^/s, power-law index *n* = 0.38, die width *b* = 360 mm and land height *h*_s_ = 1.5 mm).

**Figure 7:**
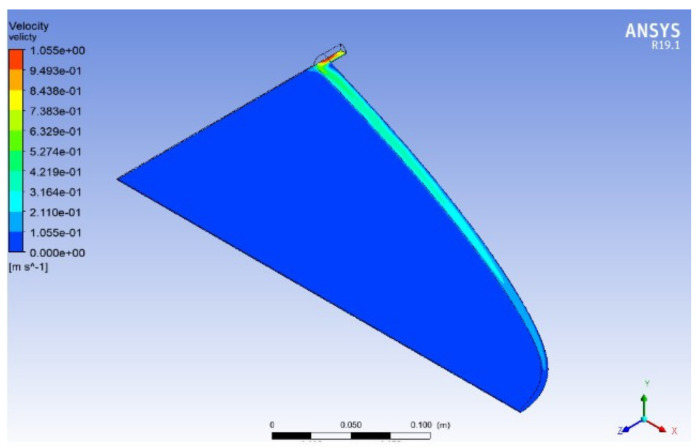
Velocity distribution of an optimized coat-hanger die using the proposed model for a power-law fluid with a circular manifold (power-law index *n* = 0.38, flow rate *Q_0_* = 5 × 10^−5^ m^3^/s, die width *b* = 360 mm and land height *h_s_* = 1.5 mm).

Figure 6 and Figure 7 show the velocity distribution at the center plane for the Winter–Fritz and the proposed design methods based on the geometric parameters shown in Figure 5, respectively. As shown in Figure 5b, the proposed method gives a larger manifold radius compared to the Winter–Fritz method. As Figure 7 shows, due to the larger cross-sectional area, the proposed method gives more uniform velocity distribution in the manifold compared to the Winter–Fritz method (Figure 6). In addition, *y*(*x*) of the proposed method (shown in Figure 5a) results in a smoother velocity distribution at the edge of the die.

For a quantitative comparison, velocity variance that is defined by Equation (24) is adapted. Velocity variances (*φ*) for the Winter–Fritz and the proposed methods are 0.00116 and 0.00078, respectively. The velocity distribution shows a 32.9% [(0.00116–0.00078)/0.00116] improvement in the proposed method.

### 3.4. Design of a Carreau-Yasuda Fluid

For other rheological models, two relations between flow rate and pressure drop for both the manifold and the slit are required. In the literature, different relations are available for this purpose [23,24,25,26]. Sochi, based on the Weissenberg-Rabinowitsch-Mooney-Schofield (WRMS) method, gives a relation for pressure-flow rate and shear rate. The Carreau–Yasuda model is defined as follows:

(32) $$\mu =\mu_{\infty }+\left(\mu_{0}-\mu_{\infty }\right){\left(1+\lambda^{2}\gamma^{2}\right)}^{\frac{n-1}{2}}=\mu_{\infty }+\delta {\left(1+\lambda^{2}\gamma^{2}\right)}^{\frac{n^{\prime }}{2}}$$

For a Carreau–Yasuda fluid flowing in a circular duct:

(33) $$Q=\frac{\pi R^{3}I_{c}}{\tau_{w}^{3}}$$

where *I_c_* and *τ_w_* are given by: [26]

(34) $$\begin{array}{ll} I_{c} & =\int_{0}^{\dot{\tau_{w}}}\gamma \tau^{2}d\tau =\int_{0}^{\dot{\gamma_{w}}}\left[\gamma^{4}\mu^{3}\frac{d\mu }{d\gamma }+\mu^{3}\gamma^{3}\right]d\gamma \\ & =\frac{\delta^{3}\left[3\lambda^{4}\left(3n^{\prime 2}+5n^{\prime }+2\right)\gamma_{w}^{4}-3n^{\prime }\lambda^{2}\gamma_{w}^{2}+2\right]{\left(1+\lambda^{2}\gamma_{w}^{2}\right)}^{3n^{\prime }/2}}{3\lambda^{4}\left(9n^{\prime 2}+18n^{\prime }+8\right)}\\ & +\frac{\mu_{i}\delta^{2}\left[\lambda^{4}\left(2n^{\prime 2}+5n^{\prime }+3\right)\gamma_{w}^{4}-n^{\prime }\lambda^{2}\gamma_{w}^{2}+1\right]{\left(1+\lambda^{2}\gamma_{w}^{2}\right)}^{n^{\prime }}}{2\lambda^{4}\left(n^{\prime }+1\right)\left(n^{\prime }+2\right)}\\ & +\frac{\mu_{i}^{2}\delta \left[\lambda^{4}\left(n^{\prime 2}+5n^{\prime }+6\right)\gamma_{w}^{4}-n^{\prime }\lambda^{2}\gamma_{w}^{2}+2\right]{\left(1+\lambda^{2}\gamma_{w}^{2}\right)}^{n^{\prime }/2}}{\lambda^{4}\left(n^{\prime }+2\right)\left(n^{\prime }+4\right)}+\frac{\mu_{i}^{3}\gamma_{w}^{4}}{4}-(\frac{2\delta^{3}}{3\lambda^{4}\left(9n^{\prime 2}+18n^{\prime }+8\right)}\\ & +\frac{\mu_{i}\delta^{2}}{2\lambda^{4}\left(n^{\prime }+1\right)\left(n^{\prime }+2\right)}+\frac{2\mu_{i}^{2}\delta }{\lambda^{4}\left(n^{\prime }+2\right)\left(n^{\prime }+4\right)}) \end{array}$$

where only the shear rate at the wall *γ_w_* is unknown. By definition, shear stress and shear rate are related by *τ = µ*(*γ*)*γ*. Momentum balance at wall gives the following:

(35) $$\tau_{w}=\mu \left(\gamma_{w}\right)\gamma_{w}=\frac{R\Delta P}{2L}$$

Due to the non-linearity of Equation (35), a numerical method is required for the shear rate at the wall (*γ_w_*). In a similar way as Sochi provided, the following relation for the flow of a Carreau-Yasuda fluid in a slit with a width of W and thickness of *H* is given:

(36) $$Q=\frac{2WH^{2}I_{pp}}{\tau_{w}^{2}}$$

(37) $$I_{pp}=\int_{0}^{\tau_{w}}\gamma \tau d\tau =\int_{0}^{\gamma_{w}}\left[\gamma^{3}\mu \frac{d\mu }{d\gamma }+\mu^{2}\gamma^{2}\right]d\gamma \;=\frac{\mu_{i}^{2}\gamma_{w}^{3}}{3}+2\mu_{i}\delta (\frac{\sqrt{\pi }\Gamma \left(-\frac{n^{\prime }}{2}-\frac{3}{2}\right)}{4\lambda^{3}\Gamma \left(-\frac{n^{\prime }}{2}\right)})+2\mu_{i}\delta \gamma_{w}^{n^{\prime }}(\frac{{\left(\lambda^{2}\right)}^{\frac{n^{\prime }}{2}}\gamma_{w}^{3}}{n^{\prime }+3}+\frac{{\left(\lambda^{2}\right)}^{\frac{n^{\prime }}{2}-1}n^{\prime }\gamma_{w}}{2n^{\prime }+2}+\frac{{\left(\lambda^{2}\right)}^{\frac{n^{\prime }}{2}}\left(n^{\prime }-2\right)n^{\prime }}{8\lambda^{4}\left(n^{\prime }-1\right)\gamma_{w}}\;+\frac{{\left(\lambda^{2}\right)}^{\frac{n^{\prime }}{2}}\left(n^{\prime }-4\right)\left(n^{\prime }-2\right)n^{\prime }}{48\lambda^{6}\left(n^{\prime }-3\right)\gamma_{w}^{n^{\prime }}})\;+\mu_{i}\delta n^{\prime }(\frac{\sqrt{\pi }\left(\Gamma \left(-\frac{n^{\prime }}{2}-\frac{3}{2}\right)\Gamma \left(1-\frac{n^{\prime }}{2}\right)-2\Gamma \left(-\frac{n^{\prime }}{2}-\frac{1}{2}\right)\Gamma \left(1-\frac{n^{\prime }}{2}\right)+2\Gamma \left(\frac{1}{2}-\frac{n^{\prime }}{2}\right)\Gamma \left(-\frac{n^{\prime }}{2}\right)\right)}{4\lambda^{3}\Gamma \left(1-\frac{n^{\prime }}{2}\right)\Gamma \left(-\frac{n^{\prime }}{2}\right)})\;+\mu_{i}\delta n^{\prime }\gamma_{w}^{n^{\prime }}(\frac{\lambda^{n^{\prime }}\gamma_{w}^{3}}{n^{\prime }+3}+\frac{\lambda^{n^{\prime }-2}\left(n^{\prime }-2\right)\gamma_{w}}{2\left(n^{\prime }+1\right)}+\frac{\lambda^{n^{\prime }}\left(n^{\prime }-4\right)\left(n^{\prime }-2\right)}{8\lambda^{4}\left(n^{\prime }-1\right)\gamma_{w}}\;+\frac{\lambda^{n^{\prime }}\left(n^{\prime }-6\right)\left(n^{\prime }-4\right)\left(n^{\prime }-2\right)}{48\lambda^{6}\left(n^{\prime }-3\right)\gamma_{w}^{3}}+\frac{\lambda^{n^{\prime }}\left(n^{\prime }-8\right)\left(n^{\prime }-6\right)\left(n^{\prime }-4\right)\left(n^{\prime }-2\right)}{348\lambda^{8}\left(n^{\prime }-5\right)\gamma_{w}^{5}})\;+\delta^{2}(\frac{\sqrt{\pi }\Gamma \left(-n^{\prime }-\frac{3}{2}\right)}{4\lambda^{3}\Gamma \left(-n^{\prime }\right)})+\delta^{2}\gamma_{w}^{2n^{\prime }}(\frac{{\left(\lambda^{2}\right)}^{n^{\prime }}\gamma_{w}^{3}}{2n^{\prime }+3}+\frac{{\left(\lambda^{2}\right)}^{n^{\prime }-1}n^{\prime }\gamma_{w}}{2n^{\prime }+1}+\frac{{\left(\lambda^{2}\right)}^{n^{\prime }}\left(n^{\prime }-1\right)n^{\prime }}{2\lambda^{4}\left(2n^{\prime }-1\right)\gamma_{w}}\;+\frac{{\left(\lambda^{2}\right)}^{n^{\prime }}\left(n^{\prime }-2\right)\left(n^{\prime }-1\right)n^{\prime }}{6\lambda^{6}\left(n^{\prime }-3\right)\gamma_{w}^{3}})\;+\delta^{2}n^{\prime }(\frac{\sqrt{\pi }\left(\Gamma \left(-n^{\prime }-\frac{3}{2}\right)\Gamma \left(1-n^{\prime }\right)-2\Gamma \left(-n^{\prime }-\frac{1}{2}\right)\Gamma \left(1-n^{\prime }\right)+2\Gamma \left(\frac{1}{2}-n^{\prime }\right)\Gamma \left(-n^{\prime }\right)\right)}{4\lambda^{3}\Gamma \left(1-n^{\prime }\right)\Gamma \left(-n^{\prime }\right)})\;+\delta^{2}n^{\prime }\gamma_{w}^{2n^{\prime }}(\frac{{\left(\lambda^{2}\right)}^{n^{\prime }}\gamma_{w}^{3}}{2n^{\prime }+3}+\frac{{\left(\lambda^{2}\right)}^{n^{\prime }-1}\left(n^{\prime }-1\right)\gamma_{w}}{2n^{\prime }+1}\;+\frac{{\left(\lambda^{2}\right)}^{n^{\prime }}\left(n^{\prime }-2\right)\left(n^{\prime }-1\right)}{2\lambda^{4}\left(2n^{\prime }-1\right)\gamma_{w}}+\frac{{\left(\lambda^{2}\right)}^{n^{\prime }}\left(n^{\prime }-3\right)\left(n^{\prime }-2\right)\left(n^{\prime }-1\right)}{6\lambda^{6}\left(2n^{\prime }-3\right)\gamma_{w}^{3}}+\frac{{\left(\lambda^{2}\right)}^{n^{\prime }}\left(n^{\prime }-4\right)\left(n^{\prime }-3\right)\left(n^{\prime }-2\right)\left(n^{\prime }-1\right)}{24\lambda^{8}\left(22n^{\prime }-5\right)\gamma_{w}^{5}})$$

Similarly, shear rate at wall is given by:

(38) $$[\mu_{i}+\delta (1+\lambda^{2}\gamma_{w}^{2}{)}^{\frac{n^{\prime }}{2}}]\gamma_{w}=\frac{H\Delta P}{L}$$

**Figure 8:**
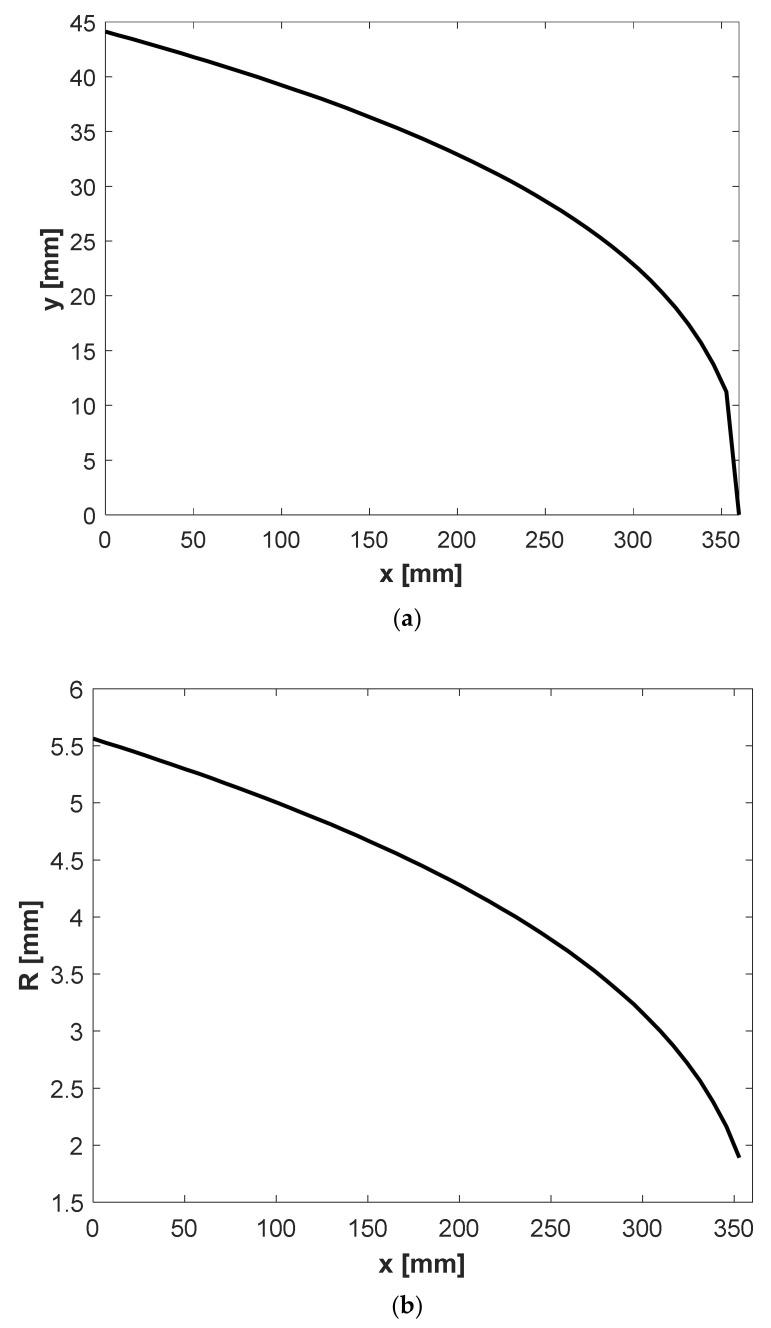
(**a**) Change of design parameter *y*(*x*) with respect to *x* and (**b**) change of the manifold radius *R* with respect to *x* for Carreau–Yasuda polypropylene (parameters in Table 2) using the proposed model with a circular manifold.

With relations provided in this section, a design for the extrusion die of a Carreau–Yasuda fluid can be given by Equation (9). Figure 8 depicts the radius and *y*(*x*) of Carreau fluid in a circular manifold for *λ* = 2.5 s, *n* = 0.75, *µ_i_* = 0.009 Pa s and *µ_0_* = 0.17 Pa s.

### 3.5. Design of a Cross Fluid

A similar methodology can be applied for a Cross fluid. A Cross fluid is defined as follows:

(39) $$\mu =\mu_{\infty }+\frac{\mu_{0}-\mu_{\infty }}{1+\lambda^{m}\gamma^{m}}$$

**Figure 9:**
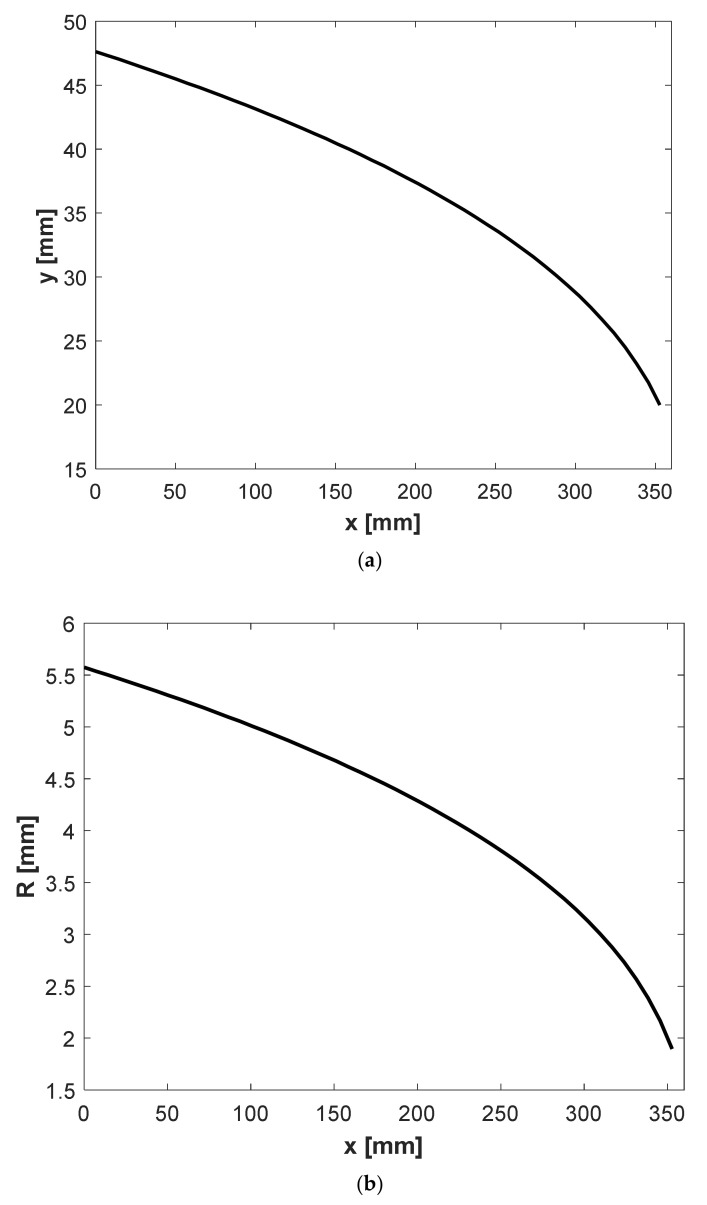
(**a**) Change of design parameter *y*(*x*) with respect to *x* and (**b**) change of manifold radius *R* with respect to *x* for a Cross polypropylene (parameters in Table 1) using the proposed model with a circular manifold.

For the cross fluid, the Weissenberg-Rabinowitsch-Mooney-Schofield (WRMS) method described by Sochi gives no analytical solution. Therefore, a numerical integration was used for Equations (19) and (22). Figure 9 depicts the radius and *y*(*x*) of a Cross fluid in a circular manifold for *m* = 0.5, *µ_0_* = 0.15 Pa s, *µ_i_* = 0.009 Pa s and *λ* = 7.9 s. In both cases, the flow rate at entry (*Q_0_*) and die width (*b*) are 5 × 10^−5^ m^3^/s and 360 mm, respectively.

**Figure 10:**
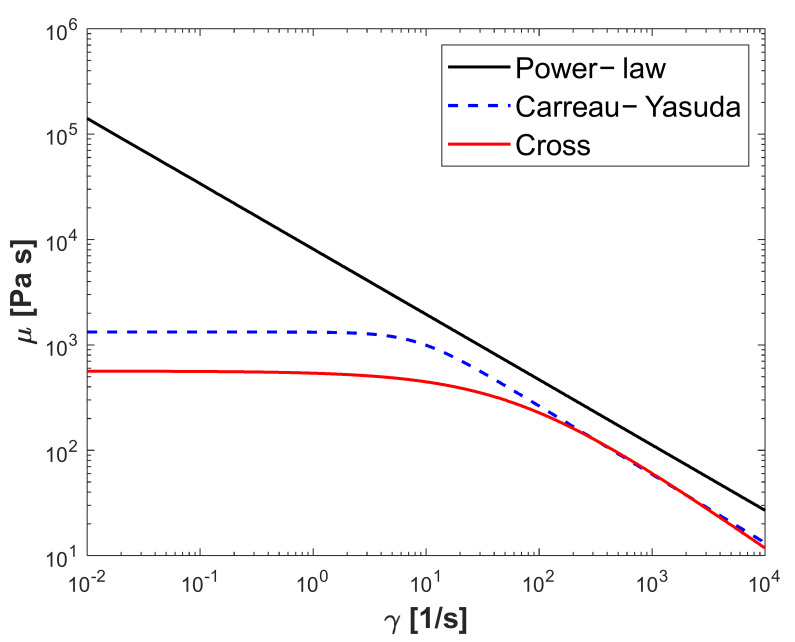
Shear rate versus the apparent viscosity of polypropylene for power-law, Cross and Carreau–Yasuda rheology models [19].

In the last three subsections, the proposed method was used for designing the die for power-law, Carreau–Yasuda and Cross fluids. Polypropylene (PP) as an extrusion fluid is used for this study. Rheological models and the corresponding parameters of polypropylene are adapted from Rudolph [19] and are shown in Table 1. Figure 10 shows a comparison of apparent viscosity based on these models. An effort to design for a PP polymer using these three rheological models is performed. The parameters of these models for PP are given in Table 2 and shown in Figure 3. Both designs based on the flow equations of the Carreau–Yasuda and Cross rheological model converge to the same design as the power-law fluid, as shown in Figure 5a,b. Winter and Fritz [3] concluded that their design only depends on the power-law index and is independent of the power-law consistency factor. For a given operational condition, wall shear rates are approximately 800–900 s^−1^. The slope of apparent viscosity versus shear rate in the logarithmic scale is the representative power-law index factor of the rheological model. As it is clearly shown in Figure 10, all rheological models of power-law, Carreau–Yasuda and Cross have a same representative power-law index, in the range of 800–900 s^−1^. As a result, it can be concluded that the proposed method is independent of all rheological parameters except the power-law index shown as n or m for power-law, Carreau–Yasuda and Cross models.

### 3.6. Desgin of a Power-Law Fluid with Rectangular Manifold

Two cases for a rectangular manifold are considered: constant manifold width (*W*) and constant manifold width to die width (*W*/*b*). The geometry for constant width proposed by Winter and Fritz is given by Equations (40) and (41):

(40) $$H\left(x\right)=h\sqrt{\frac{b-x}{Wf_{p}\left(x\right)}}$$

(41) $$y\left(x\right)=2W\sqrt{\frac{b-x}{W}-1}$$

In the case of a constant *a = W/b* ratio, the following equations are given by Winter and Fritz:

(42) $$H\left(x\right)=h{\left[\left(b-x\right)/\left(af_{p}h\right)\right]}^{\frac{1}{3}}$$

(43) $$W\left(x\right)=aH\left(x\right)={\left[a^{2}h^{2}\left(b-x\right)/f_{p}\right]}^{\frac{1}{3}}$$

(44) $$y\left(x\right)=\frac{3Bb}{2}\left[\frac{\sqrt{1+g\left(x\right)}}{g\left(x\right)}-\frac{1}{2}\ln\frac{\sqrt{1+g\left(x\right)}-1}{\sqrt{1+g\left(x\right)}+1}\right]$$

where *g*(*x*) and *B* are given by:

(45) $$g\left(x\right)={\left[{\left(H\left(x\right)/h\right)}^{2}-1\right]}^{-1}$$

(46) $$B=\frac{ahf_{p}}{b}$$

**Figure 11:**
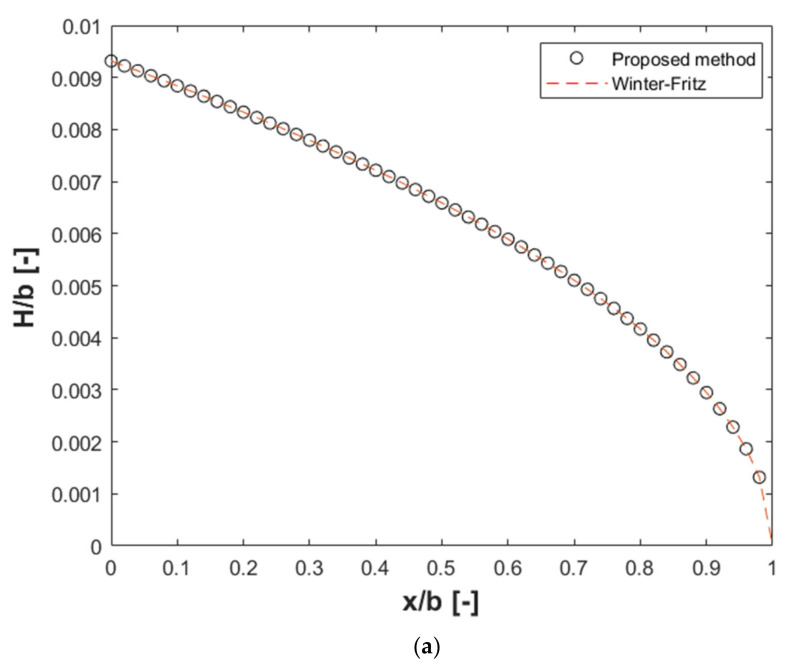

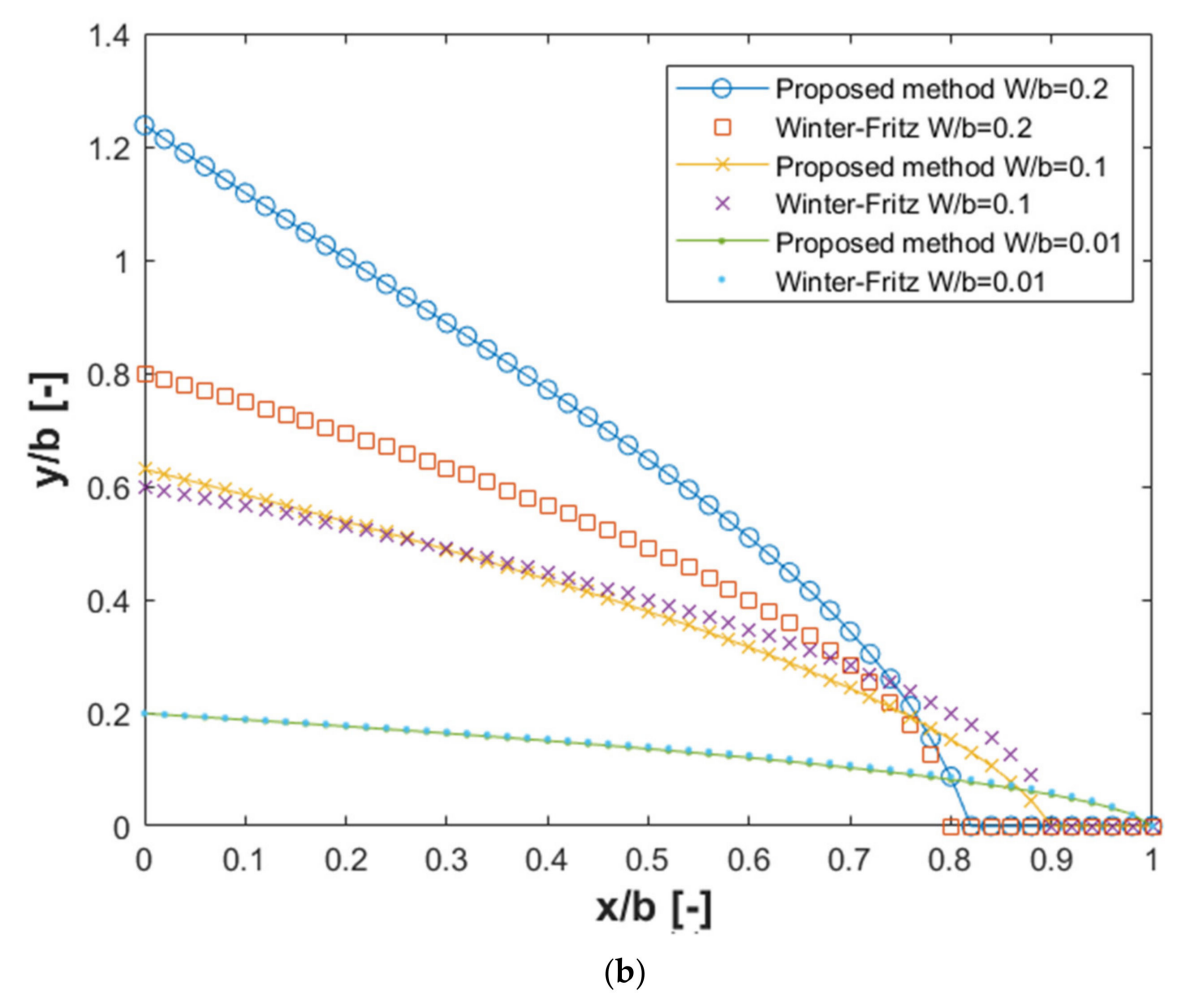
(**a**) Change of normalized design parameter *H* with respect to normalized *x* and (**b**) change of normalized design parameter *y* with respect to normalized *x* for power-law polypropylene (parameters in Table 1) having a rectangular manifold with constant *W/b* ratio, (*W/b* = 0.01, 0.1 and 0.2, N = 50).

**Figure 12:**
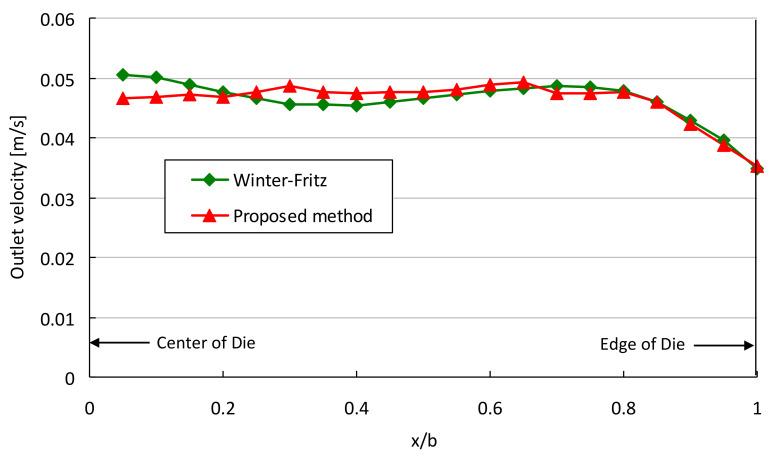
Comparison of velocity distribution obtained from CFD at the slit exit of die for a power-law fluid with *W/b* = 0.19.

Figure 11 shows the comparison of manifold height and die land for the case of constant *W*. Figure 12 shows the comparison of the velocity at the exit obtained from CFD for both designs. A comparison of velocity variance defined in Equation (24) gives values of 0.005 and 0.0039 for the Winter–Fritz and the proposed methods, respectively. This result shows a 21.5 percent improvement in the velocity variance. By comparing velocity variances, it is clear that the die designed with a circular manifold is approximately three-fold more efficient in terms of velocity uniformity than the die designed with a rectangular manifold.

## 4. Conclusions

In this study, a general rheology network method for the design of sheeting extrusion dies in the polymer processing industry is proposed based on the Winter–Fritz constant wall shear-rate conditions and conservation laws of fluid flows in the network. Design parameters based on this method for dies with both circular- and rectangular-shaped manifolds are presented and validated as examples of demonstrations of the method. Computational fluid dynamics (CFD) was adapted as a means for comparison of the presented design models in this study with the purely analytical model of Winter and Fritz. The CFD results show 32.9% and 21.5% improvement in the velocity distribution for dies with circular and rectangular manifolds, respectively, over similar dies designed by other methods. In addition, a method for calculation of wall-flow rates/pressure drops is presented based on the work of Sochi [26]. Since this method is inherently flexible, it can be easily extended to other rheological models. It is demonstrated that the proposed method can be broaden and applied to other rheological models such as Carreau–Yasuda and Cross models, which are more accurate at low shear rates. Thus, the proposed method utilizes constant shear-rate constraint of the Winter–Fritz for power-law and virtually for any other rheology model. Furthermore, it can be extended to take into account temperature effects, such as viscous dissipation, as well as the elastic deformation of the die slit, with some modifications. The proposed method is a fast and computationally efficient method for the first design iteration of sheeting extrusion dies with constant shear rates, as CPU time for every design calculation takes less than a few seconds on a PC (Intel Core i7 2.6 GHz).

The uniform velocity distribution leads to less material loss, which is both economically and environmentally desirable. The proposed semi-analytical design method, which was developed for the fast and accurate pre-design of coat hanger dies with applications in the film and sheeting industries, can be further extended to blow molding, wire-coating and cable sheathing, and the textile and food industries.

## Figures and Tables

**Table 1:** Rheology models and corresponding parameters [20].

Rheology Model	Equation	Parameters
Power-law	$\eta =k{\dot{\gamma }}^{n-1}$	*k* = 8.125 × 10^3^ Pa s^n^ *n* = 0.38
Carreau–Yasuda	$\eta =\mu_{\infty }+\left(\mu_{0}-\mu_{\infty }\right){\left(1+\lambda^{2}\gamma^{2}\right)}^{\frac{n-1}{2}}$	*µ*_0_ = 1326 Pa s^n^ *µ*_∞_ = 0 *λ* = 0.12 s *n* = 0.35
Cross	$\mu =\mu_{\infty }+\frac{\mu_{0}-\mu_{\infty }}{1+\lambda^{m}\gamma^{m}}$	*µ*_0_ = 564.4 Pa s^n^ *µ*_∞_ = 0 *λ* = 0.017 s *m* = 0.749

**Table 2:** Input design parameters of the model.

Parameter	Value
Flow rate in the entry of the die, *Q_0_*	5 × 10^−5^ m^3^/s
Land height, *h_s_*	1.5 mm
Total die width at exit, *b*	360 mm

## Data Availability

The data presented in this study are available on request from the corresponding author.

## Nomenclature

*a*	Ratio of manifold width to half die thickness (*W*/*b*)
*b*	Half die thickness [m]
*B*	Parameter in Winter–Fritz equation
*f_p_*	Shape factor in Winter–Fritz model
*g*	Parameter in Winter–Fritz equation
*H*	Manifold height [m]
*h_s_*	Slit height [m]
*I*	Identity tensor
*I_c_*	Definite integral definition for circular duct in Equation (22) [Pa^3^ s^−1^]
*I_pp_*	Definite integral definition for parallel plates Equation (19) [Pa^2^ s^−1^]
*k*	Power-law consistency factor
*m*	Cross model parameter
*n*	Power-law index
*N*	Number of manifold and slit segments
*Q_0_*	Flow rate in entry [m^3^/s]
*Q_m_*	Flow rate in the manifold [m^3^/s]
*Q_s_*	Flow rate in the slit [m^3^/s]
*R*	Radius of manifold [m]
*v*	Velocity [m/s]
*φ*	Velocity variance
*W*	Width of channel [m]
*x*	Coordinate along die [m]
*y*	Distance between manifold and die outlet [m]
Δ*P_m_*	Incremental pressure drops in manifold [Pa]
Δ*P_s_*	Incremental pressure drops in slit [Pa]
*µ*	Apparent viscosity [Pa s]
*µ_0_*	Rheological parameter in Cross and Carreau–Yasuda models [Pa s]
*µ_∞_*	Rheological parameter in Cross and Carreau–Yasuda models [Pa s]
*γ_m_*	Shear rate in manifold [1/s]
*γ_s_*	Shear rate in slit [1/s]
*γ_w_*	Shear rate at wall [1/s]
*ζ*	Coordinate in direction of manifold [m]
*λ*	Rheological parameter in Cross and Carreau–Yasuda models [s]
*τ*	Shear stress [Pa]
*τ_w_*	Shear stress at wall [Pa]
*φ*	Velocity variance [m^2^/s^2^]
*dP_m_*	Differential pressure drops in manifold [Pa]
*dP_s_*	Differential pressure drops in slit [Pa]
